# Type IX Superior Labrum Anterior and Posterior Lesion in a Professional Football Player: A Rare Pattern of Shoulder Instability in a Non-throwing Athlete

**DOI:** 10.7759/cureus.34753

**Published:** 2023-02-07

**Authors:** Edgar Amorim, Pedro Maganinho, Diogo Rodrigues-Gomes, Sérgio Rodrigues-Gomes, Nuno Sevivas

**Affiliations:** 1 Physical Medicine and Rehabilitation, Hospital de Braga, Braga, PRT; 2 Radiology, Centro Hospitalar Universitário do Porto, Porto, PRT; 3 Human Performance Department, Sport Lisboa e Benfica, Lisbon, PRT; 4 Radiology, Espregueira-Mendes Sports Center, Porto, PRT; 5 Orthopedics and Traumatology, Centro Hospitalar do Médio Ave, Braga, PRT

**Keywords:** return to sport, type ix slap lesion, sports medicine, sports rehabilitation, slap repair, slap diagnosis, slap tear, anterior shoulder instability

## Abstract

Anterior shoulder instability is the most frequent type of glenohumeral instability, especially among young athletes. Superior labral anterior-posterior (SLAP) injuries involve the superior glenoid labrum where the long head of the biceps tendon (LHBT) inserts. There is still some debate regarding the pathogenesis, clinical presentation, and treatment of these lesions. We report a clinical case of an 18-year-old male professional football player with a rare type IX SLAP lesion. Given the recurrence of instability after prior nonoperative management, surgical treatment was seen as the best option, and a pan-labral arthroscopic repair suture anchor fixation was performed. Three months after undergoing a personalized postoperative rehabilitation program, he was able to return to full sport with the same competitive level, and no recurrent instability or other symptoms were reported throughout the 18-month follow-up period.

## Introduction

The glenohumeral joint is the most frequently dislocated joint of the human body [1]. In trying to control the propensity for instability, the shoulder has multiple stabilizers, both static and dynamic, that are responsible to restrain humeral translation and can be injured during daily activities [2]. Labral lesions are widely described in the literature, however, their imaging diagnosis is challenging and their classification and treatment are not always consensual [3-5]. 

Superior labral anterior and posterior (SLAP) lesions involve the superior glenoid labrum where the long head of the biceps tendon (LHBT) inserts, and are a common finding in shoulder arthroscopies, with an occurrence rate of up to 26% [6]. They can extend into the tendon, involve the glenohumeral ligaments, or extend into other quadrants of the labrum often causing shoulder pain, dysfunction, blocking, and instability symptoms [3]. The sports-relative activity appears to be the greatest risk factor for these lesions, particularly if associated with repetitive movements above the shoulder level [6,7]. 

Initially classified by Snyder into four distinct types, additional categories of SLAP injuries and specific subtypes were then added by other authors. A total of ten types of SLAP lesions have been described [3, 8-10]:

- Type I: fraying with intact biceps tendon;

- Type II: tear of the bicipito-labral complex; 

- Type IIa: tear of the bicipito-labral complex with more anterior extension;

- Type IIb: tear of the bicipito-labral complex with more posterior extension;

- Type IIc: tear of the bicipito-labral complex with anterior and posterior extension;

- Type III: bucket-handle tear of superior labrum with biceps tendon intact;

- Type IV: bucket-handle tear of superior labrum extension to biceps tendon;

- Type V: Bankart lesion in continuity with type II SLAP tear;

- Type VI: anterior or posterior flap tear of the superior labrum with biceps tendon stripping;

- Type VII: tear extends into middle glenohumeral ligament;

- Type VIII: superior labral tear with posteroinferior labral tear;

- Type IX: superior labral tear with extensive anterior and posterior extension/pan-labral tear;

- Type X: superior labral tear with extension to the rotator interval.

A type IX SLAP lesion is a particularly rare abnormality that is defined as a pan-labral injury with detachment of the superior labrum and biceps anchor that extends across the entire circumference of the glenoid [10].

We report a clinical case of a type IX SLAP lesion in a professional football player after a single traumatic event with shoulder dislocation. 

## Case presentation

**Figure 1:**
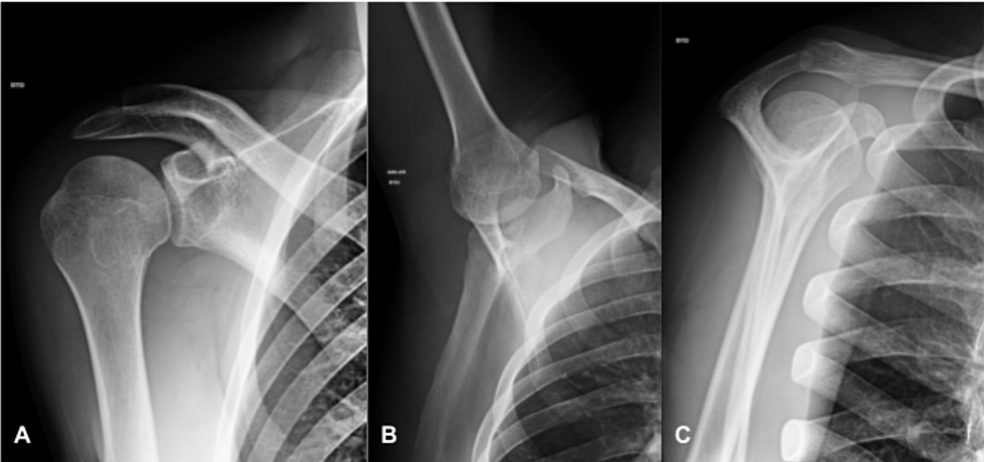
Post-reduction radiographic study of the right shoulder A - anteroposterior view; B - axillary view; C - Y view. All studies showed normal alignment of the bone structures, with no fractures or other bone lesions. No relevant soft tissue damage was apparent.

An 18-year-old male professional footballer playing as a striker, with no previous history of upper limb traumatic injuries, had a fall with his right arm being forced into excessive abduction and external rotation and felt a severely painful and popping sensation in his right shoulder. At the initial on-field clinical assessment, he was supporting his injured arm with the hand of the contralateral side in a slightly abducted position and presented a loss of shoulder round contour with a prominent humeral head and acromion, compatible with an anterior acute dislocation. After ruling out neurovascular injuries and a pre-reduction radiographic study was obtained, a successful manual reduction maneuver was performed. A post-reduction radiograph was obtained and showed the integrity and normal position of all bone structures (Figure 1).

**Figure 2:**
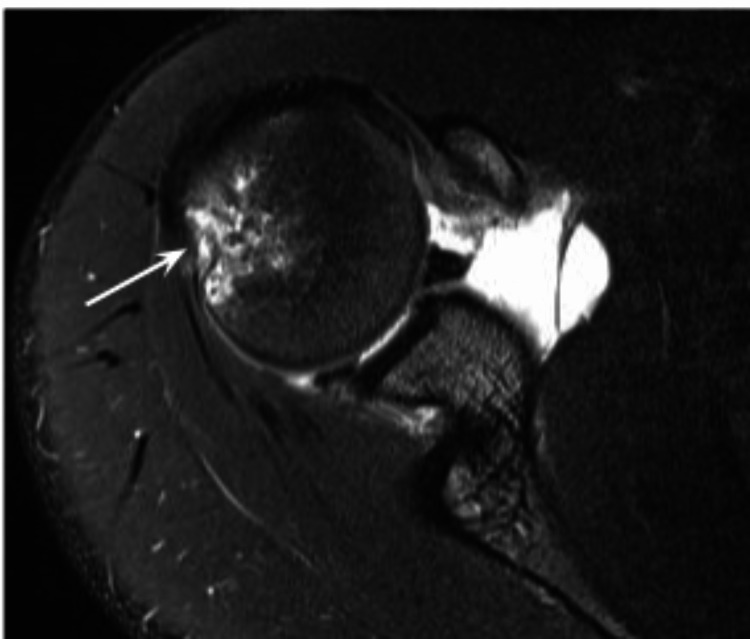
Axial fat-suppressed proton density-weighted magnetic resonance arthrogram (MRA) image of the right shoulder Bony defect compatible with Hill-Sachs lesion (arrow) and bone contusion on the posterolateral aspect of humeral head.

**Figure 3:**
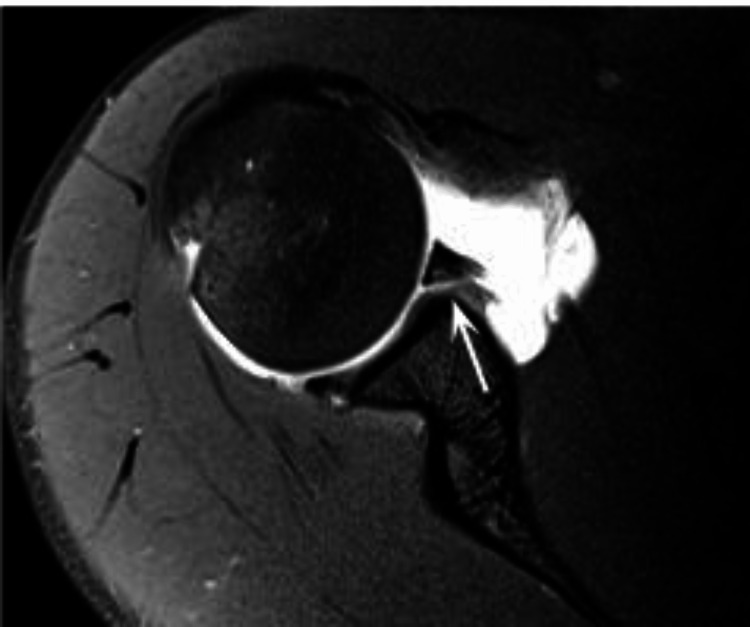
Axial fat-suppressed T1-weighted MRA image of the right shoulder The* *anteroinferior labrum has become detached from the glenoid (arrow) and is associated with a torn scapular periosteum - Bankart Lesion.

**Figure 4:**
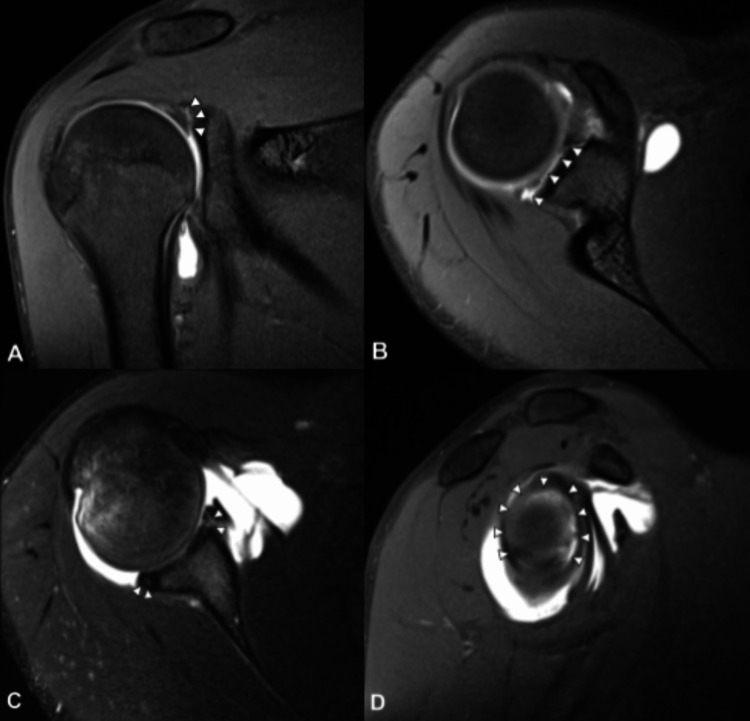
Proton density-weighted fat-suppressed MRA image of the right shoulder A. Coronal proton density-weighted fat-suppressed MRA image showing intra-articular contrast column dissecting between the avulsed glenoid labrum and glenoid cartilage (arrowheads). B, C, and D. Axial and sagittal proton density-weighted fat-suppressed MRA images demonstrating the progression of the anteroinferior labral tear into the anterior, posterosuperior, posterior, and posteroinferior labrum (7 o’clock to 5 o’clock) or circumferential or pan-labral lesion - type IX superior labral anterior and posterior (SLAP) lesion.

In order to search for other soft-tissue injuries (e.g. cartilaginous, ligamentous, or muscle/tendinous), a magnetic resonance arthrogram (MRA) was then performed. Besides an on-track non-engaging Hill-Sachs (Figure 2) and cartilaginous Bankart (Figure 3) lesions, it revealed a concomitant superior labrum tear with extensive anterior and posterior extension compatible with a type IX SLAP lesion (Figure 4).

A conservative approach was followed and a successful return to play with the same competitive level was achieved after 8 weeks. Four months after the initial injury, the patient had a second episode of anterior shoulder dislocation after a fall on the outstretched arm during a training session. The imaging investigation showed the exact same findings as previously described. Given the failure of prior nonoperative treatment, the likelihood of recurrence, and the fact that it was near the end of the competition season, surgical treatment was considered the best option. Intraoperatively, the diagnostic arthroscopy confirmed the presence of a Bankart cartilage lesion and the desinsertion of the superior, anterior, and posterior glenoid labrum (type IX SLAP injury). An arthroscopic labral repair with eight suture anchors was then performed. 

Postoperatively, the athlete underwent a 4-week period in a sling with the shoulder positioned in internal rotation and slightly anterior to the coronal plane aiming to protect the repair while also minimizing biceps contraction. During this phase, the main goals were to control pain and inflammation, optimize tissue healing, initiate shoulder range of motion (ROM), and improve dynamic stabilization. Gentle pendulum and active-assistive ROM exercises were first introduced in week 3 post-surgery. Active biceps contraction was avoided for 4 weeks to prevent stress on the healing biceps-labral complex. After the sling was removed, more aggressive ROM exercises, scapular stabilization, and pain-free submaximal rotator cuff and deltoid isometrics were gradually included. Proprioception and neuromuscular stability were emphasized to normalize the scapulohumeral rhythm. Isotonic strengthening exercises starting with light-resistance and high-repetition were incorporated during week 6, first in isolation, and later in combination with proprioceptive neuromuscular facilitation patterns in both open and closed kinetic chain. At week 8, the patient began a structured running progression program and started specific football drills with subsequent more complex field workouts. He was able to gradually return to performance with the team, firstly avoiding contact activities, and then progressing until the previous competitive level was achieved after 12 weeks. During the 18-month period of follow-up, no recurrent instability or other symptoms have been reported.

## Discussion

Anterior instability is by far the most frequent type of glenohumeral instability, especially among young athletes [1,11]. In contrast to other contact sports like American football and rugby, where shoulder injuries often affect the acromioclavicular joint, most severe football shoulder injuries tend to affect the glenoid labrum [12]. Despite a frank relatively lower incidence than lower limb injuries, a 4-year period longitudinal study of English professional football teams has demonstrated that shoulder injuries are becoming more common and severe over time [13].

The exact pathophysiology of SLAP tears remains uncertain. Multiple injury mechanisms have been proposed, both acute and chronic, including direct compression load through a fall on an outstretched arm with the shoulder in abduction and slight flexion, traction to the arm, and repetitive overhead activities [5, 14]. 

In the acute setting, the anterior dislocated shoulder has a characteristic appearance with a prominent humeral head and there is a loss of the normal rounded contour over the deltoid. The initial examination must include a neurovascular examination of the upper limb, with special emphasis on the axillary nerve function [15]. Ideally, plain radiographs should be obtained prior to and post-reduction to assess for concomitant bone lesions and document acceptable reduction. MRA is considered the gold standard for the assessment of instability and preoperative workup for shoulder ligaments and labral injuries [11,15,16]. A high incidence of associated injuries is frequent, such as the detachment of the labrum to the anteroinferior glenoid margin, known as classic Bankart lesion, the most common labral injury. Hill-Sachs defect resulting from a compression fracture on the posterolateral aspect of the humeral head caused by the impaction of the anteriorly dislocated humeral head with the anterior glenoid rim is also frequently found [5,6,11].

Athletes with shoulder injuries frequently have several coexisting injuries with similar clinical presentations and there is no definitive test at the physical examination that allows discerning between them. Despite several provocative tests for SLAP tears having been proposed, their diagnostic value is inconsistent [5]. In a meta-analysis by Meserve et al, the O’Brien active compression test was identified to be the most sensitive test for SLAP tears with a sensitivity of 47 to 78%, and Speed’s test had the highest specificity of 67 to 99% [17].

After an anterior shoulder dislocation, it is important to discuss the risks and benefits of the different treatment options. The in-season athlete may undergo conservative treatment with proper rehabilitation recognizing the risk of recurrent instability [11,18]. Although glenohumeral instability might be a significant injury in field players, it usually does not preclude them from returning to high-level competition. Persistent pain and recurrent instability are more frequent among goalkeepers and can negatively affect their performance in specific position tasks like diving or throwing the ball [11,12]. Contraindications to non-operative in-season management include failure of prior conservative treatment, engaging Hill-Sachs lesion, bony Bankart >20%, humeral avulsion of glenohumeral ligament lesions, and previous failed stabilization surgery [2,11].

There are few case descriptions regarding the treatment of type IX SLAP injuries, but all agree in recommending arthroscopic treatment for debridement, labral reinsertion with suture anchors, or biceps tenodesis or tenotomy [19]. Tokish et al studied the outcomes of a prospective cohort of patients with circumferential lesions who were managed with pan-labral arthroscopic repair suture anchor fixation. All shoulders improved significantly in subjective and objective outcome measures and all returned to their preinjury activity level, including eight high-performance contact athletes, with an overall failure rate of 15% [20].

Rehabilitation after surgical intervention typically involves temporary immobilization using a sling and subsequent initial pendulum exercises and isometric deltoid contractions when the pain subsides. To protect the surgical repair, it is important to restrict movements that may place torsional stresses including active shoulder flexion, extension, abduction, external rotation as well as elbow flexion and supination. After 3-4 weeks, active and passive range of motion of the glenohumeral may be introduced, and around 6 weeks post-surgery, progressive strengthening of the rotator cuff and scapular stabilizers may be initiated. It is advisable to avoid active shoulder flexion beyond 90º and forcefully contract the biceps for approximately 6 weeks [10]. Finally, progressive sports-specific activities can be individualized based on the mechanism of injury, functional demands, and activity level.

## Conclusions

Complex shoulder injuries involving the glenoid labrum must be considered in the workup of patients who present with shoulder instability, particularly in athletes, even after a single acute event. Despite some debate, operative treatment may be deemed the preferred treatment option in contact athletes, and the risks involved in a conservative treatment or delayed surgical stabilization should be properly discussed.

In this case, it was possible to demonstrate a successful approach to a particularly rare type of labral lesion in a professional football player that returned to his prior level of sports performance.
